# Cortical florbetapir-PET amyloid load in prodromal Alzheimer’s disease patients

**DOI:** 10.1186/2191-219X-3-43

**Published:** 2013-06-03

**Authors:** Laure Saint-Aubert, Emmanuel J Barbeau, Patrice Péran, Federico Nemmi, Celine Vervueren, Helene Mirabel, Pierre Payoux, Anne Hitzel, Fabrice Bonneville, Raluca Gramada, Mathieu Tafani, Christian Vincent, Michele Puel, Sophie Dechaumont, Francois Chollet, Jeremie Pariente

**Affiliations:** 1 INSERM, Imagerie Cérébrale et Handicaps Neurologiques UMR 825, Centre Hospitalier Universitaire de Toulouse, Place Dr Baylac, Pavillon Baudot, Toulouse CEDEX 9 31059, France; 2 Université de Toulouse III, UPS, Imagerie Cérébrale et Handicaps Neurologiques UMR 825, Centre Hospitalier Universitaire de Toulouse, Toulouse 31062, France; 3 Centre de Recherche Cerveau et Cognition (CerCo), CNRS, Université de Toulouse III, UPS, Toulouse CEDEX 9, 31059, France; 4 Service de Neurologie, Pôle Neurosciences, Centre Hospitalier Universitaire de Toulouse, Place Dr Baylac, Toulouse CEDEX 9, 31059, France; 5 Service de Médecine Nucléaire, Pôle Imagerie, Centre Hospitalier Universitaire de Toulouse, Place Dr Baylac, Toulouse CEDEX 9, 31059, France; 6 Service de Neuroradiologie, Pôle Imagerie, Centre Hospitalier Universitaire de Toulouse, Place Dr Baylac, Toulouse CEDEX 9, 31059, France; 7 Laboratoire de Biologie Cellulaire et Cytologie, Pôle Biologie, Centre Hospitalier Universitaire de Toulouse Purpan, Toulouse CEDEX 9, 31059, France

**Keywords:** Alzheimer's disease, Florbetapir, Amyloid, Imaging, Memory

## Abstract

**Background:**

Florbetapir (AV-45) has been shown to be a reliable tool to assess amyloid load in patients with Alzheimer's disease (AD) at demential stages. Longitudinal studies also suggest that AV-45 has the ability to bind amyloid in the early stages of AD. In this study, we investigated AV-45 binding and its relation with cognitive performance in a group of patients at the prodromal stage of Alzheimer's disease, recruited according to strict inclusion criteria.

**Methods:**

We recruited patients at the prodromal stage of AD and matched control subjects. AV-45 binding was assessed using an innovative extraction method allowing quantifying uptake in the cortex only. AV-45 uptake was compared between groups in the precuneus, posterior cingulate, anterior cingulate, and orbito-frontal regions. Correlations between AV-45 uptake and cognitive performance were assessed.

**Results:**

Twenty-two patients and 17 matched control subjects were included in the study. We report a significant increase of cortical AV-45 uptake in the patients compared to the control subjects in all regions of interest. Specific correlations were found within the patient group between mean global amyloid cortical load and cognitive performance in three different memory tests.

**Conclusions:**

These findings suggest that at the prodromal stage of AD, memory decline is linked to an increase of cortical β-amyloid load.

## Background

In the past decade, it has been recognized that Alzheimer's disease (AD) has a clinical stage before dementia occurs, a stage now known as prodromal. The diagnosis of prodromal AD according to research criteria [1,2] relies on an objective cognitive impairment, most often a memory decline, as assessed by a neuropsychological evaluation, and one or more of the following specific features: medial temporal lobe atrophy on structural magnetic resonance imaging (MRI), temporo-parietal hypometabolism on positron-emission tomography (PET) scan using ^18^F-fluorodeoxyglucose (FDG), or markers of amyloid pathology. As recently published in the new AD and mild cognitive impairment (MCI) criteria, amyloid biomarkers tend to have an increased weight in the diagnosis [3,4]. Hence, amyloid biomarkers are nowadays embedded in the diagnostic criteria, not only for AD-related dementia, but also for the prodromal stage of AD (see Dubois et al. [2]). Several studies have shown that the use of amyloid biomarkers may drastically modify the accuracy of AD diagnosis. In these studies, *in vivo* amyloid pathology is assessed using cerebrospinal fluid (CSF) or through specific ligands by PET imaging. CSF amyloid-beta (Aβ) biomarkers have now been widely studied [5]. New ratios between Aβ42, Aβ40, tau, and phospho-tau and the use of new concentration cutoffs [5,6] appear to be reliable tools to detect AD at an early stage [7]. Numerous studies have been conducted using PET to bind amyloid plaques *in vivo*[8,9]. ^18^F-florbetapir (AV-45) ligand shows increased uptake in the cortex of AD patients compared to control subjects [10,11]. AV-45 shows good correlations with post-mortem lesions [12,13]. AV-45 has shown good consistency with clinical examination in patients having the genetic forms of AD [14,15] as well as in sporadic forms at the demential stage [11]. A few studies have assessed AV-45 uptake in patients with MCI [10,16-18]. Fleisher et al. showed a significant increase of AV-45 uptake in MCI compared to healthy subjects [10]. Doraiswamy et al. reported that MCI patients with visually positive AV-45 images showed clinical worsening and tended to convert to dementia at a higher rate [16]. However, MCI patients may have pathologies other than AD or even no pathology at all. Florbetapir has never been studied in a population of prodromal AD patients who were characterized according to research criteria [1].

A problem regarding AV-45 binding is that it is suggested to bind in a non-specific manner to the white matter [12]. Up to now, only a few studies have attempted to deal with this issue using masking techniques [19,20]. These studies showed that more methodological developments are required.

AV-45 binding is also related to lower episodic memory performance in clinically normal older individuals [21]. So far, no correlation has been reported between cognitive performance and AV-45 uptake in AD patients at the prodromal stage.

In this study, our aim was to investigate the profile of cortical AV-45 binding, using a new method, and its relation to cognitive performance in a group of AD patients at the prodromal stage recruited according to strict inclusion criteria in comparison with a group of matched cognitively normal subjects.

## Methods

### Participants

All participants gave their written informed consent. This study was approved by the local ethics committee (Comité de Protection des Personnes Sud-Ouest et Outre-Mer I) and the French Agency for Safety and Security of Medical Devices (Agence Française de Sécurité Sanitaire des Produits de Santé, reference A90605-58).

For this study, patients at the prodromal stage of AD [2] over 65 years old were recruited. They all came from the outpatient memory clinic (Neurology Department, University Hospital, Toulouse, France). Matched control subjects were recruited among patients' relatives or by recruitment posting in public places.

### Pre-inclusion assessment

Patients were invited to enroll in the study if they presented a memory complaint dating from more than 6 months, had no concomitant neurologic or psychiatric disease history, and were not affected by any clinically significant pathology that could explain their memory complaint. Patients then underwent the following:

• *Pre-inclusion neuropsychological assessment*: Autonomy in daily life was assessed using the Clinical Dementia Rating (CDR) scale. Anterograde verbal memory was assessed using the Free and Cued Selective Reminding Test (FCSRT) [22].

• *Brain MRI*: Brain MRI was performed in all participants using a Philips 3-T imager (Intera Achieva, Philips, Best, The Netherlands). A high-resolution anatomical image, using a three-dimensional (3D) T1-weighted sequence (in-plane resolution 1 × 1 mm, slice thickness 1 mm, 160 contiguous slices) and a T2-weighted sequence (reconstructed resolution 0.45 × 0.45 × 3 mm^3^, 43 slices) were obtained. Two independent neuroradiologists with extensive experience (FB and RG), blind to clinical information, examined all sequences at inclusion and rated them for both medial temporal lobe atrophy on the 3D T1 sequence using the Scheltens scale [23] and white matter changes on the T2-weighted images using the Fazekas and Schmidt (F&S) scale [24]. Atrophy was assessed for the two hemispheres separately. Inter-rater agreement was estimated by calculating Cohen's kappa coefficient (*κ*) and its 95% confidence interval (CI).

• *FDG-PET scan*: Scans were performed on a Biograph 6 TruePoint Hirez (Siemens Medical Solutions, Munich, Germany) hybrid PET/computed tomography (CT) scanner (3D detection mode, producing images with 1 × 1 × 1.5 mm voxels and a spatial resolution of approximately 5 mm full width at half maximum at the field of view center). Cerebral emission scans began 20 min after the injection of 1.85 MBq/kg weight of FDG on average and lasted for 10 min. Both CT and PET scans were acquired. PET data were corrected for partial volume effects. Two independent nuclear medicine physicians with extensive experience in reading FDG-PET scans (PiP and AH), blind to clinical information, examined all FDG-PET scans at inclusion. A three-point scale was used for rating FDG-PET profiles (0 = normal, 1 = temporo-parietal hypometabolism suggestive of AD, 2 = other). Inter-rater agreement was estimated by calculating *κ* and its 95% CI.

• *CSF biomarker sampling*: CSF samples were obtained by a lumbar puncture in the patient group. The samples were centrifuged for 10 min at 1,500 rpm at 4°C to remove cells, aliquoted to 0.4-mL samples in polypropylene tubes, and stored at -80°C until analysis. CSF biomarker levels of total tau (T-Tau), phospho-tau (P-Tau), Aβ42, and Aβ40 were measured using a sandwich ELISA method (Innogenetics, Gent, Belgium) according to the manufacturer's instructions. We also calculated ratios derived from single biomarkers, including the Innotest Amyloid Tau Index (IATI), combining Aβ42 and T-Tau concentrations as follows: IATI = Aβ42 / (240 + (1.18 × T-Tau)), and the Aβ42/Aβ40 ratio.

Control subjects underwent the same neuropsychological assessment, MRI, and FDG-PET scans as the patients.

### Inclusion criteria

Following these examinations, patients were given the diagnosis of prodromal AD [2] and included in the present study if they met the following criteria: CDR = 0.5, sum of the three free recalls ≤17/48, and/or sum of the three free and cued recalls ≤40/48 on the FCSRT [25], and one or more of the following features:

• Scheltens score for medial temporal lobe atrophy >1 in at least one hemisphere for at least one visual rater [23].

• Temporo-parietal hypometabolism pattern on cerebral FDG-PET scan (score = 1 for at least one visual rater).

• P-Tau ≥ 60 pg/mL and IATI ≤ 0.8. In the case of an ‘ambiguous’ profile (P-Tau < 60 pg/mL or IATI > 0.8), Aβ42/Aβ40 was calculated and a score < 0.045 was considered as compatible with AD diagnosis [6,26].

• Patients with significant white matter T2 hyperintensities (F&S score > 2 for at least one visual rater) were excluded.

Control subjects were included if they had no memory complaint, no neurological or psychiatric disease history, or no first-degree relatives with AD. They were excluded if they showed significant white matter hyperintensities on their T2-weighted MR images (F&S score > 2 for at least one visual rater) or any cognitive impairment on the pre- or post-inclusion neuropsychological assessment (test scores below -2 standard deviations according to the norms).

Patients and control subjects were then assessed with detailed neuropsychological evaluation and AV-45-PET.

### Post-inclusion assessment

#### Neuropsychological assessment

A comprehensive battery of tests was used for all participants. Global cognitive state was assessed using the Mini-Mental State Examination (MMSE), while the 4-Instrumental Activities of Daily Living test was used to assess daily-life autonomy. The following cognitive domains were assessed: visual memory (Rey-Osterrieth Complex Figure Test (RCFT) [27], DMS48 [28]), semantic memory (Weschler Adult Intelligence Scale (WAIS) information subtest [27], TOP 12 faces [29]), verbal working memory (WAIS-III digit span [27]), praxies (RCFT copy, praxies evaluation protocol [30]), language (DO80 - a French confrontation naming test [31]), executive functions (phonemic and semantic verbal fluencies [27], Trail Making Test (TMT) [27], Frontal Assessment Battery (FAB) [32], Stroop [27]), gnosia (Benton Facial Recognition Test [27]), and attention (TMT, Symbol Digit Modalities Test [27]). Anxiety and depression were also assessed using the State-Trait Anxiety Inventory (Y-A form) [33] and Beck Depression Inventory [27], respectively.

#### AV-45-PET scan

All participants underwent a second PET scan using the AV-45 amyloid marker. Scans were performed on the same PET/CT scanner as for FDG-PET, using identical reconstruction parameters. Cerebral emission scans began 50 min after an injection of 3.7 MBq/kg weight of AV-45 and lasted for 20 min. Both CT and PET scans were acquired. PET data were corrected for partial volume effects.

#### ApoE alleles

This analysis was performed using blood samples from the patient group only. All these examinations were spread over three different appointments, scheduled within 3 months maximum.

### Statistical analysis

#### Neuropsychological assessment

Intergroup comparisons were performed using the Mann–Whitney statistical test.

#### MRI

Cortical morphology differences between the two groups were assessed using a voxel-based morphometry (VBM) method on Statistical Parametric Mapping version 8 (SPM8; Wellcome Trust Centre for Neuroimaging, London, UK) software running on MATLAB (Mathworks Inc., Sherborn, MA, USA). For each subject, 3D T1 sequence was normalized to the SPM8 template, then segmented to isolate gray matter and white matter partitions, and modulated for deformations. The resulting modulated gray matter maps were then smoothed (8 × 8 × 8 mm) and pooled by group for statistical inter-group comparison using voxel-based analysis (threshold for significance *p* < .001, uncorrected; cluster = 20 voxels).

#### FDG- and AV-45-PET global uptake

For both ligands, uptake differences between groups were assessed using a voxel-based analysis on SPM8 (threshold for significance *p* < .001, uncorrected; cluster = 20 voxels). FDG-PET scans were whole-brain-normalized using a PET template from SPM8, smoothed (8 × 8 × 8 mm), and pooled by group for statistical comparison. AV-45-PET scans were whole-brain-normalized using a template from Avid [34], smoothed (8 × 8 × 8 mm), and pooled by group for statistical comparison.

#### AV-45 regional uptake values in the cortex only

Due to AV-45 non-specific binding reported in the white matter [11], regional mean standardized uptake values (SUVs) were calculated from the cortex only as follows: For each subject, CT scan obtained during AV-45-PET acquisition was first linearly registered onto the MRI anatomical T1 image using FSL software. The obtained transformation matrix was then applied to the AV-45 image of the subject so that AV-45 image was in the T1 space. Gray matter mask from T1 segmentation (see MRI statistical analysis) was binarized using a 0.3 threshold and applied to the AV-45 image. An Anatomical Automatic Labeling [35] template was also registered onto each individual T1 space using the inverse of the transformation matrix obtained, registering the individual T1 onto the Montreal Neurological Institute (MNI) space. Then measures of regional cortical AV-45 mean SUV were conducted for each subject using an in-house MATLAB script. Mean global cortical SUV was calculated as well as SUV from specific regions of interest (ROIs): orbito-frontal, anterior cingulate, posterior cingulate, and precuneus. These regions were selected as they have shown different AV-45 binding in studies comparing AD patients to control subjects [9-11,36]. SUVs were then normalized (SUVr) to whole cerebellar mean uptake (vermis excluded) and pooled by group for statistical comparison. Inter-group regional uptake difference was assessed using a non-parametric Mann–Whitney statistical test (threshold for significance *p* < .05), and the Bonferroni-Holmes correction for multiple comparisons was applied.

#### AV-45 SUVr correlation analyses

Correlations were investigated in the patient group between mean global cortical AV-45 SUVr and cognitive performance scores at memory tests: delayed free recall and delayed total recall subtests of the FCSRT [22] (verbal anterograde memory), recall of the RCFT [27] (visual anterograde memory), the DMS48 set 2 score (visual memory), the WAIS information subtest (semantic memory), the naming score, and the total score at the TOP 12 [29] (an innovative test assessing semantic memory about the life of 12 celebrities from their face). Correlations between relevant AV-45 SUVr and CSF marker concentrations were also investigated in patients. The Spearman non-parametrical test was used (threshold for significance *p* < .05).

## Results

A total of 34 patients and 25 control subjects enrolled in the study. Among them, 22 patients and 17 control subjects satisfied the inclusion criteria and completed the whole protocol. There was no significant difference in age, sex, or level of education between the patients and the control groups (Table 1).

**Table 1:** Population description and performance at neuropsychological assessment

	**Prodromal patients**	**Control subjects**	***p *value**
*n*	22	17	NA
Age	72.4 ± 5.0	69.9 ± 4.8	.110
Gender	12 M/10 F	7 M/10 F	.408
Level of education	11.3 ± 2.7	12.8 ± 3.3	.163
Disease duration	3.8 ± 3.6	NA	NA
ApoE genotype	2 E4/E4	NA	NA
	9 E3/E4		
	5 E3/E3		
	3 E2/E3		
Daily-life autonomy			
CDR scale	0.5 ± 0.0	0.0 ± 0.0	<.001*
Anterograde verbal memory			
FCSRT sum of free recalls (/48)	11.6 ± 5.9	32.2 ± 4.6	<.001*
FCSRT sum of free + cued recalls (/48)	28.7 ± 11.9	46.6 ± 1.9	<.001*
Global cognitive state			
MMSE	25.7 ± 1.4	28.4 ± 0.7	<.001*
Depression scale			
Beck (/39)	3.0 ± 2.6	2.7 ± 2.6	.666
Anxiety scale			
Y-A (/80)	34.3 ± 8.9	3.2 ± 6.0	.830
Anterograde visual memory			
DMS48 set 1 (/48)	41.0 ± 6.0	46.5 ± 2.0	.003*
DMS48 set 2 (/48)	40.0 ± 7.0	45.9 ± 2.2	.014*
Rey complex figure memory (/36)	9.2 ± 6.6	19.4 ± 6.1	.002*
Semantic memory			
Information subtest (WAIS) (/28)	13.8 ± 5.5	20.1 ± 6.2	.014*
TOP 12 faces version: global score (/96)	74.8 ± 6.7	84.6 ± 6.0	.003*
TOP 12 faces version: naming (/12)	5.2 ± 3.4	9.3 ± 2.2	.006*
Short-term memory			
WAIS-III digit span: forward	5.6 ± 1.4	5.4 ± 1.0	1.763
Working memory			
WAIS-III digit span: backward	4.0 ± 1.2	4.6 ± 0.9	.514
Language			
DO80 (/80)	78.1 ± 3.8	79.4 ± 1.2	.549
Praxies			
Rey complex figure copy (/36)	34.4 ± 1.9	34.6 ± 2.0	1.258
Speed processing			
Digit-symbol test (/90)	34.1 ± 12.6	52.8 ± 10.2	<.001*
Executive functions			
Phonemic verbal fluency: letter (P)	19.6 ± 8.1	22.6 ± 6.1	.674
Semantic verbal fluency: ‘animal’ category	21.7 ± 7.3	31.8 ± 7.4	.012*
TMT B time	170.3 ± 85.3	94.1 ± 38.4	.014*
Interference time on the Stroop test	108.1 ± 45.1	51.6 ± 31.9	.002*
FAB (/18)	15.1 ± 2.5	17.1 ± 0.8	.098
Gnosia			
Benton Facial Recognition (/58)	46.0 ± 3.3	48.1 ± 3.5	.476

CDR scale, Clinical Dementia Rating scale; FCSRT, Free and Cued Selective Recall Reminding Test; NA, not applicable. *p < .05 (significant difference on the Mann–Whitney test).

### Fulfillment of the inclusion criteria for prodromal AD

On imaging data, MRI visual assessment revealed temporal atrophy (Scheltens score > 1) in 17 patients out of 22 (left hippocampus atrophy: *κ* = 0.465 with CI 0.15 to 0.78, right hippocampus atrophy: *κ* = 0.767 with CI 0.46 to 1.0). Visual assessment of FDG-PET scans revealed temporo-parietal hypometabolism in 14 patients out of 22 (*κ* = 1). Of the 22 patients, 20 had a lumbar puncture for CSF biomarkers; the two remaining patients did not give their consent for CSF sampling. Eighteen of the 20 patients showed a pathological CSF profile. One was classified as normal, and one was considered ambiguous, with P-Tau < 60 pg/mL despite IATI ≤ 0.8 (no values available for Aβ40 concentration for this patient; details on individual profiles are available in Additional file 1).

When the neuropsychological assessment, MRI, FDG, and CSF profiles were combined, 3 patients had two markers in favor of prodromal AD, 11 had three markers, and 8 had all four markers consistent with prodromal AD.

### Inter-group comparisons of the inclusion data

#### Neuropsychological assessment

Patients showed significant memory and executive function impairment compared to the control group. No significant deficit was reported regarding other cognitive fields. Patients were neither depressed nor anxious (Table 1).

#### MRI

Compared to the control group, patients showed significant atrophy, mainly in the hippocampal regions but also in the frontal and parietal regions, on VBM analysis (*p* < .001, uncorrected; Additional file 2A).

#### FDG-PET

The analysis was carried out between 22 patients and 16 control subjects. One control subject was excluded from the analysis due to unusable scan data. On voxel-based analysis, the patient group revealed a large hypometabolism in the parietal and also the temporal and frontal lobes compared to the control group (*p* < .001, uncorrected; Additional file 2B).

### AV-45 imaging

#### Whole brain profile of AV-45 binding

On voxel-based whole brain analysis, the patient group showed higher AV-45 uptake in the precuneus compared to the control group (*p* < .001, uncorrected; Additional file 2C).

#### Regional profile of AV-45 binding

**Figure 1:**
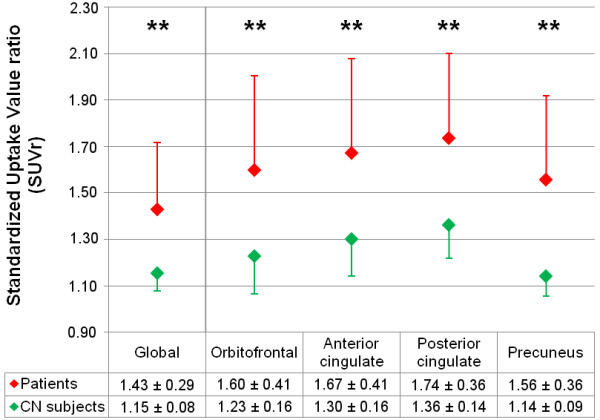
**AV-45-PET imaging uptake. **Regional-to-cerebellum standard uptake values (SUVr) for AV-45 marker in the global cortex (left side of the vertical line) and specific regions of interest (right side of the vertical line) are shown with associated standard deviations. Red diamonds, patients; green diamonds, control subjects. **p < .01 (significant inter-group difference on the Mann–Whitney test, Bonferroni-Holmes-corrected).

SUVr analysis in gray matter ROIs showed a significant AV-45 increased uptake in the patient group compared to the control group in the global cortex as well as in the precuneus, anterior and posterior cingulate, and orbito-frontal regions (Figure 1; mean values are shown in Table 2).

**Table 2:** Regional AV-45 uptake

	**AV-45-PET values**		
	**Patients’ mean SUVr (±sd)**	**Control subjects’ mean SUVr (±sd)**	***p *value**
Global cortex	1.43 (±.29)	1.15 (±.08)	.002**
Orbito-frontal	1.60 (±.41)	1.23 (±.16)	.001**
Anterior cingulate	1.67 (±.41)	1.30 (±.16)	.003**
Posterior cingulate	1.74 (±.36)	1.36 (±.14)	.002**
Precuneus	1.56 (±.36)	1.14 (±.09)	.002**

AV-45 mean regional-to-cerebellum standard uptake values (SUVr) with associated standard deviation (sd) are mentioned for the global cortex and specific regions of interest for the two groups. **p < .01 (significant inter-group difference on the Mann–Whitney test, Bonferroni-Holmes-corrected).

#### AV-45 SUVr correlation analyses in the patient group

**Figure 2:**
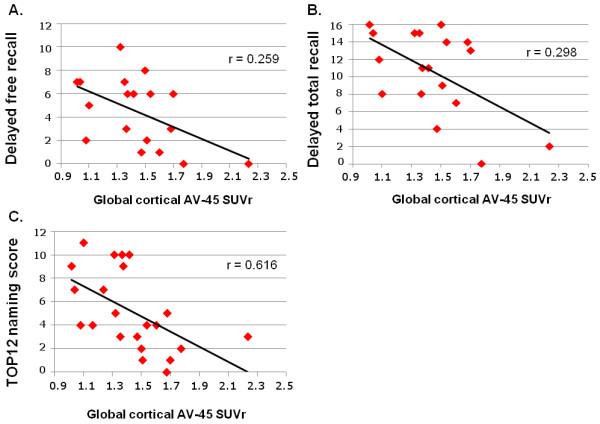
**Correlation between mean global cortical AV-45 standardized uptake value ratio (SUVr) and memory performance. **At (**A**) the delayed free recall of the FCSRT (max = 16), (**B**) the delayed total recall of the FCSRT (max = 16), and (**C**) the naming subtest of the TOP 12 semantic test (max = 12).

Performance on the delayed free recall (*r* = -.504, *p* = .017) and delayed total recall (*r* = -.553, *p* = .008) subtests of the FCSRT as well as the naming score at the TOP 12 semantic test (*r* = -.616, *p* = .002) correlated negatively with global SUVr (Figure 2). Of note, AV-45 uptake did not correlate with age, and no correlations were found between AV-45 uptake and CSF concentrations in the patient group.

## Discussion

In this study, we used selective criteria to recruit patients presenting with prodromal AD [2]. Despite having fully preserved autonomy, they showed cognitive impairment, brain atrophy, and hypometabolism typical of AD compared to control subjects as well as CSF profile typical of AD. Nineteen of the 22 patients showed a typical profile of AD on at least three of these four markers. Patients consistently showed increased AV-45 uptake compared to control subjects on both whole brain analysis and cortical analysis using regions of interest. In the group of patients, we also identified significant correlations between cortical AV-45 uptake and memory performance. To our knowledge, the present study is the first to assess AV-45 cortical uptake in a population of prodromal AD patients [2].

### Profile of cerebral AV-45 uptake

The AV-45 PET marker has been shown to reliably assess cerebral amyloid load in patients with AD [37], showing an increased uptake in AD patients, most reliably reported in frontal and parietal regions [12]. Consistently, we found increased cortical uptake in our patients in the whole cortex as well as in specific regions of interest: the orbitofrontal lobe, the anterior and posterior cingulate, and the precuneus, in agreement with previous studies [10,11,19]. Similar binding patterns have been shown in numerous studies on MCI patients using the ^11^C-PiB amyloid biomarker [38,39].

### AV-45 specificity

AV-45 has been reported as showing non-specific binding in the white matter. In a recent study comparing AV-45 and PiB fixation in AD patients at the demential stage, Wolk et al. reported greater overlaps in uptake between AD and control groups with AV-45 than with PiB [9]. The authors proposed that AV-45 ROI SUVr included local non-specific white matter uptake, reducing the specificity of the findings compared to PiB in patients. To avoid such issue, our AV-45 images were masked to exclude non-gray matter voxels from the analyses. To our knowledge, only two other studies using AV-45 considered the gray matter only [19,20]. Rodrigue et al. used AV-45 images of young subjects as white matter masks for their elderly subjects [20]. In a recent study, La Joie et al. excluded the white matter from the analysis using a unique mask applied on normalized images in the MNI space for all participants [19]. In the present study, we developed an optimized method to quantify AV-45 binding in the cortex only, using each subject's MRI white matter segmentation as a mask on their respective AV-45 image. Our results thus apply to AV-45 binding in the cortex only.

### Relation between AV-45 uptake and cognitive performance

In this work, we focused on the correlation between AV-45 uptake in ROIs and memory performance in the patient group. A correlation was found with cognitive performance in memory tests assessing verbal, visual, and semantic memory. These results imply that memory performance declines as the cerebral β-amyloid load increases. Different authors have suggested that the amyloid load would reach a plateau at the onset of the symptomatic stage [40,41]. Accordingly, no correlation should be expected with cognitive performance. However, we did identify correlations with memory performance, as did one recent study using AV-45 [16] and another using PiB [42], both in MCI patients. Longitudinal studies assessing PiB binding in MCI reported a cognitive decline along with an increase ligand uptake [43,44]. Overall, this suggests that the amyloid load may reach its maximum at a more variable stage of the disease than usually reported, an idea deserving further consideration. We did not find a correlation between AV-45 uptake and CSF in patients, in contrast to other PiB studies [45]. This is possibly due to the fact that patients were partly selected according to their pathological CSF profile.

## Conclusions

This study on prodromal AD patients recruited according to strict inclusion criteria revealed, using an optimized method of quantification on the cortex only, increased cortical amyloid load compared to cognitively normal subjects. The amyloid load correlated with memory decline. The AV-45 marker appears to be a promising tool for the early, pre-demential diagnosis of AD, in particular when focusing the analyses on gray matter uptake.

## Competing interests

Pr. P. Payoux received honoraria from serving on the scientific advisory board of Lilly. Pr. F. Chollet is currently a consultant for ‘Institut de recherche Pierre Fabre, France.’ Pr. J. Pariente serves as an editorial member of the Journal of Alzheimer's Disease and received grants from the abovementioned Agence Nationale de la Recherche (French National Research Agency) and the Toulouse teaching hospital for this study. All other author declare that they have no competing interests.

## Authors’ contributions

All authors have contributed to the work to some extent as follows: LSA participated in acquiring and interpreting the data, performed the statistical analysis, and drafted the manuscript. EJB conceived of the study, participated in its design and coordination, and helped draft the manuscript. PaP analyzed the imaging data and revised the manuscript. FN analyzed the imaging data. CV acquired the neuropsychological data. HM acquired the neuropsychological data. PiP analyzed and interpreted the PET imaging data. AH analyzed and interpreted the PET imaging data. FB and RG analyzed and interpreted the MRI imaging data. MT acquired the PET imaging data. CV carried out the biological sample analyses. MP and SD acquired the clinical data. FC contributed to the conception and design of the study. JP conceived of the study, participated in its design and coordination, and helped draft the manuscript. All authors read and approved the final manuscript.

## Supplementary Material

Additional file 1 **Individual profiles of patients on inclusion criteria and mean global target-to-cerebellum Standard Uptake Values (SUVr) for both groups. **‘+’ refers to the fulfillment of the criterion, and ‘-’ refers to the absence of abnormality on assessment. NA = not available. ‘ambiguous’ refers to CSF biomarkers missing the Aβ40 concentration to help determine the CSF profile. Click here for file

Additional file 2 **Inter-group imaging analyses. **Threshold for significance *p *< .001 (uncorrected). A. Cerebral atrophy of patients compared to controls. B. Hypometabolism in patients compared to controls. C. Increased AV-45 uptake in patients compared to controls. Click here for file
