# Supplementary material for: Cortical florbetapir-PET amyloid load in prodromal Alzheimer’s disease patients
Source: EJNMMI Res. 2013 Jun 3;3:43. doi: 10.1186/2191-219X-3-43 (PMC3733998; doi:10.1186/2191-219X-3-43)
Supplement: Additional file 1 — Individual profiles of patients on inclusion criteria and mean global target-to-cerebellum Standard Uptake Values (SUVr) for both groups. ‘+’ refers to the fulfillment of the criterion, and ‘-’ refers to the absence of abnormality on assessment. NA = not available. ‘ambiguous’ refers to CSF biomarkers missing the Aβ40 concentration to help determine the CSF profile. [file 2191-219X-3-43-S1.pdf]

| Patient | Medial temporal atrophy | Temporo-parietal hypometabolism | AD-like CSF biomarkers | AV-45 mean global SUVr |
|---|---|---|---|---|
| n°1 | - | + | + | 1.50 |
| n°2 | + | - | + | 2.23 |
| n°3 | + | + | + | 1.51 |
| n°4 | - | + | + | 1.42 |
| n°5 | + | - | + | 1.08 |
| n°6 | + | + | + | 1.54 |
| n°7 | + | + | + | 1.47 |
| n°8 | + | + | + | 1.32 |
| n°9 | + | - | + | 1.02 |
| n°10 | + | - | ambiguous (IATI = 0.38 ; P-Tau =0.48pg/mL) | 1.16 |
| n°11 | + | - | + | 1.37 |
| n°12 | + | + | NA | 1.68 |
| n°13 | + | + | + | 1.04 |
| n°14 | + | + | - | 1.24 |
| n°15 | + | - | NA | 1.31 |
| n°16 | - | + | + | 1.68 |
| n°17 | + | + | + | 1.70 |
| n°18 | + | + | + | 1.37 |
| n°19 | - | - | + | 1.35 |
| n°20 | + | - | + | 1.77 |
| n°21 | - | + | + | 1.10 |
| n°22 | + | + | + | 1.60 |

| CN subjects | AV-45 mean global SUVr |
|---|---|
| n°1 | 1.05 |
| n°2 | 1.16 |
| n°3 | 1.09 |
| n°4 | 1.14 |
| n°5 | 1.17 |
| n°6 | 1.35 |
| n°7 | 1.23 |
| n°8 | 1.23 |
| n°9 | 1.16 |
| n°10 | 1.14 |
| n°11 | 1.05 |
| n°12 | 1.21 |
| n°13 | 1.10 |
| n°14 | 1.20 |
| n°15 | 1.16 |
| n°16 | 1.15 |
| n°17 | 1.04 |

**Additional data 1: Individual profiles of patients on inclusion criteria and mean global target-to-cerebellum Standard Uptake Values (SUVr) for both groups.**
