# Supplementary figures and images for: Cortical florbetapir-PET amyloid load in prodromal Alzheimer’s disease patients
Source: EJNMMI Res. 2013 Jun 3;3:43. doi: 10.1186/2191-219X-3-43 (PMC3733998; doi:10.1186/2191-219X-3-43)

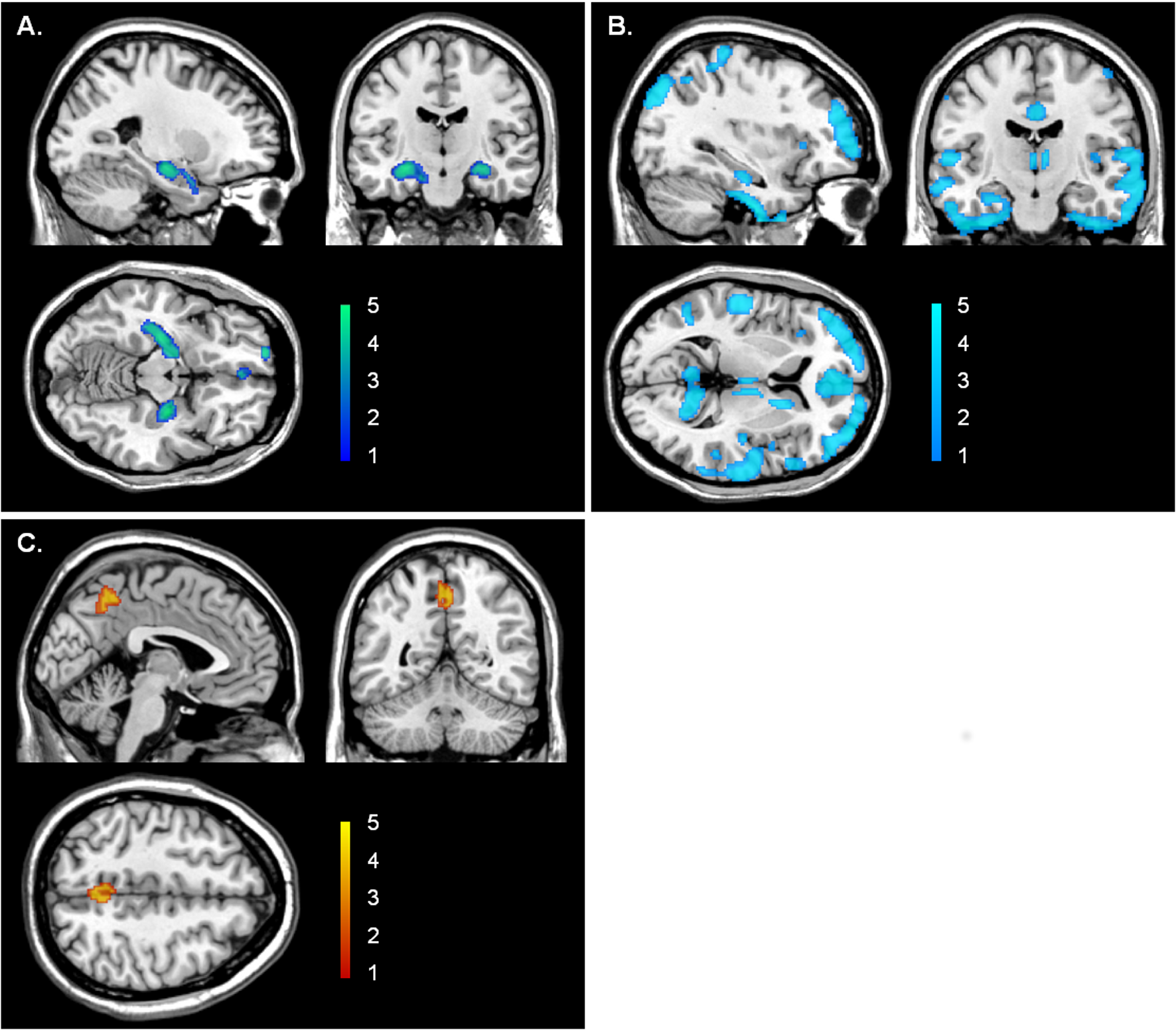

Supplement: Additional file 2 — Inter-group imaging analyses. Threshold for significance p < .001 (uncorrected). A. Cerebral atrophy of patients compared to controls. B. Hypometabolism in patients compared to controls. C. Increased AV-45 uptake in patients compared to controls. [file 2191-219X-3-43-S2.tif]
